# Tailored Microcantilever Optimization for Multifrequency Force Microscopy

**DOI:** 10.1002/advs.202303476

**Published:** 2023-10-22

**Authors:** Gourav Bhattacharya, Indrianita Lionadi, Andrew Stevenson, Joanna Ward, Amir Farokh Payam

**Affiliations:** ^1^ Nanotechnology and Integrated Bioengineering Centre (NIBEC), School of Engineering Ulster University Belfast BT15 1AP UK

**Keywords:** atomic force microscopy, dip‐coating, eigenfrequency, harmonics, microcantilever

## Abstract

Microcantilevers are at the heart of atomic force microscopy (AFM) and play a significant role in AFM‐based techniques. Recent advancements in multifrequency AFM require the simultaneous excitation and detection of multiple eigenfrequencies of microcantilevers to assess more data channels to quantify the material properties. However, to achieve higher spatiotemporal resolution there is a need to optimize the structure of microcantilevers. In this study, the architecture of the cantilever with gold nanoparticles using a dip‐coating method is modified, aiming to tune the higher eigenmodes of the microcantilever as integer multiples of its fundamental frequency. Through the theoretical methodology and simulative model, that integer harmonics improve the coupling in multifrequency AFM measurements is demonstrated, leading to enhanced image quality and resolution. Furthermore, via the combined theoretical‐experimental approach, the interplay between induced mass and stiffness change of the modified cantilever depending on the attached particle location, size, mass, and geometry is found. To validate the results of this predictive model, tapping‐mode AFM is utilized and bimodal Amplitude Modulation AFM techniques to examine and quantify the impact of tuning higher‐order eigenmodes on the imaging quality of a polystyrene‐polymethylmethacrylate (PS‐PMMA) block co‐polymer assembly deposited on a glass slide and Highly Ordered Pyrolytic Graphite (HOPG).

## Graphical abstract

Gold nanoparticles are precisely deposited on a cantilever's surface through integrated drop casting dip‐coating approach, adjusting higher eigenmodes to align with integer multiples of the first eigenmode. Through statistical and image analysis methods, it is shown this customized cantilever enhances image quality and resolution during the imaging of block co‐polymer PS‐PMMA surfaces using AFM tapping mode and bimodal amplitude modulation techniques.

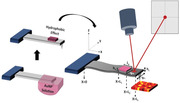

## 1 Introduction

Imaging and nanoscale characterization of the organization, structure, interfaces, and properties of the emerging engineering, functional and biological materials such as 2D, topological, quantum, hyperbolic, and carbon‐based materials^[^ ^1^, ^2^, ^3^, ^4^, ^5^ ^]^ as well as additive manufactured polymers, composites,^[^ ^6^, ^7^ ^]^ and protein complexes^[^ ^8^, ^9^, ^10^ ^]^ rely on the advanced microscopy and characterization techniques. Among microscopy techniques including optical, confocal, electron, and near‐field scanning optical and atomic force microscopies, due to its applicability in ambient conditions, ease of use, high resolution, and ability to perform both imaging and characterization, atomic force microscopy (AFM) and its modalities have become the unique remarkable technique.^[^ ^11^, ^12^ ^]^

Microcantilever is a critical element of AFM whose properties determine the speed and resolution of images as well as the identification of materials properties.^[^ ^13^, ^14^ ^]^

The principle of AFM performance is based on the deflection and/or vibration of the microcantilever utilized to probe the tip‐surface interaction. Depending on the type and range of interaction as well as the time scale of measurements, AFM can provide a diverse range of information such as atomistic and molecular structure of the surface, and physical, chemical, thermal, mechanical, electronic, and magnetic properties of specimen.^[^ ^15^ ^]^ The primary principle of AFM operation is classified into two modes of operation: contact or static and dynamic mode.^[^ ^13^ ^]^ In most modern studies, the dynamics AFM is preferred, as the microcantilever is externally vibrated to enhance sensing accuracy while better preserving the tip and the sample.^[^ ^16^ ^]^ More recently, based on the principle of dynamics mode AFM, through simultaneous excitation and detection of eigenfrequencies of microcantilevers several advanced techniques called multifrequency AFM are developed to explore more information from the materials during imaging.^[^ ^17^ ^]^ Due to the dynamic nature of advanced AFM techniques, during the nonlinear interaction between the cantilever‐tip ensemble and specimen, higher harmonics of the fundamental frequency of the vibrating cantilever emerge.^[^ ^18^, ^19^, ^20^ ^]^ The main source of these harmonics’ responses is short‐range attractive and repulsive forces as well as contact time. As a consequence, these harmonics carry plentiful information on the mechanical, physical, and chemical properties of the specimen.^[^ ^18^, ^20^ ^]^ Recent studies demonstrated the harmonics of microcantilever signal are highly sensitive to the local elasticity of the materials,^[^ ^18^, ^21^ ^]^ and higher contrast with detailed imaging were achieved from the higher harmonics of the microcantilever signal rather than its fundamental frequency.^[^ ^22^, ^23^ ^]^ Hence, using harmonic amplitude images with higher spatial resolution can open a path for fast, robust, and non‐invasive mapping of mechanical, physical, and chemical properties of advanced materials at the nanoscale.

However, the main challenge in the detection of higher harmonics signals is obtaining a reasonable signal‐to‐noise ratio (SNR).^[^ ^22^, ^24^, ^25^, ^26^, ^27^ ^]^ To enhance the higher harmonics, several efforts have been performed either by exciting the cantilever at a submultiple of its natural resonance frequency^[^ ^28^ ^]^ or by driving its torsional mode^[^ ^29^ ^]^ as well as using multifrequency AFM via coupling the eigenmodes to increase the harmonics of first eigenmode.^[^ ^30^ ^]^ Although these methods could enhance the specific harmonics of the microcantilever signal, the way the cantilever is excited affects the quality of the captured topography images which is based on the excited frequency to which the feedback controller is applied. So, rather than an excitation strategy, it may be better to optimize the microcantilever structure as a force sensor.^[^ ^31^, ^32^ ^]^ Recent studies demonstrated that changing the mass distributions of the microcantilever via either adding or removing local masses is the most convenient way to optimize the performance of the harmonics.^[^ ^33^ ^]^

Via this approach, the higher eigenmodes of the cantilever can be tuned to be an integer multiple of its fundamental frequency which leads to the enhancement of the corresponding harmonics response. Based on this methodology, Sahin et al,^[^ ^34^ ^]^ tuned the third flexural eigenmode to the 16th harmonic of the natural resonance frequency by removing the mass from the cantilever.

Felts et al. introduced a paddle structure on the cantilever to tune the ratio of the first two eigenfrequencies in a diverse integer range.^[^ ^35^ ^]^ However, in addition to design and fabrication complexity, there is a significant change in the effective stiffness of the modified cantilever especially at a small frequency ratio. Cai et al,^[^ ^36^, ^37^ ^]^ modified the standard cantilevers by varying the width of the cantilever as well as adding a step cross‐section to the cantilever. The fabrication process of these designs is rather complex and results in difficulty in the geometrical control in manufacturing.

To enhance the harmonic response of microcantilevers, following Sahin's approach,^[^ ^34^ ^]^ Zhang et al.^[^ ^27^ ^]^ tuned the frequency properties by altering the mass distribution of the cantilever by cutting a rectangular slot. However, this approach makes a hole in the cantilever which affects its dynamic response in ambient conditions and liquids especially when the cantilever is squeezed by interfacial liquid at the solid surface or when the environment is humid.

Li et al.^[^ ^38^ ^]^ and Xiang et al.^[^ ^38^ ^]^ attached a concentrated particle to the cantilever to simultaneously tailor the eigenfrequencies. However, to optimize tuning, the heavy attached particle is required. Furthermore, in their modelling approach, they just consider the changes of eigenfrequencies attributed to the added mass of the particle which leads to the decrease of eigenfrequencies while the primary mechanism of the resonant frequency change occurs through both induced mass‐change and spring constant‐change (stiffness‐change) and can lead to either increase or decrease of eigenfrequencies.^[^ ^39^ ^]^ Despite the mentioned valuable efforts, there is a lack of comprehensive understanding of the effect of the attached particle geometry and its mass, the cantilever structure and the interplay between the mass distribution and the stiffness of the cantilever in decreasing/increasing eigenfrequencies and the simultaneous tuning of the higher eigenmodes to the fundamental frequency ratio to an integer value.

In this paper, we detail our straightforward and comprehensive theoretical, numerical, and experimental methodology to model, analyse and optimize the tailored cantilevers.

Using our presented approach, we study the effect of the interplay between the induced stiffness and mass changes of the attached particle on the frequency shift and tuning the eigenfrequencies of the microcantilever. Our simulation predicts that the nature of the frequency shift (positive and negative shift) and stiffness of the cantilever depend on the location, shape, size and geometry of the attached foreign particles. Based on our theoretical and numerical findings, we modify the cantilever with gold nanoparticles using a dip‐coating method to adjust the third and fourth eigenmode to be an integer harmonic of the modified fundamental resonant frequency. Via dynamic AFM and bi‐modal AM AFM measurements, we demonstrate that the excitation of the third and fourth eigenmode of the modified cantilever can significantly enhance the spatial resolution thus providing an opportunity for a robust and non‐destructive mechanical mapping of polymer and carbon‐based materials.

## 2 Model

**Figure 1:**
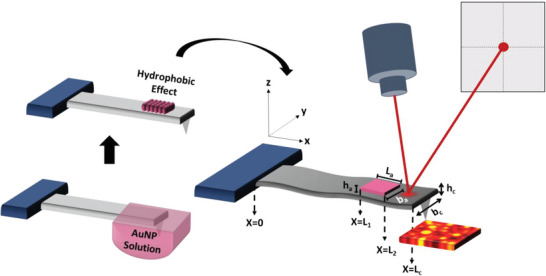
Schematic of dip coating method and modified cantilever with added mass.

For our presented analysis, the cantilever geometry as presented in **Figure** **1** is approximated as an ideal rectangular shape with an attached mass.

The cantilever is singly clamped at one end and free at the other end. The length of the cantilever is *L_c_*, width *b_c_* and thickness *h_c_* oriented with the x‐axis with flexural displacement along the z‐axis. The attached particle length is *L_a_*, width *b_a_* and thickness is *h_a_*.

By neglecting rotatory inertia and shear deformation, the flexural displacement of the cantilever with added particle obeys the following differential equation:

(1) $$\begin{array}{ccc} & & \frac{\partial^{2}}{\partial x^{2}}\left(\left(E_{c}I_{c}+E_{a}I_{a}\left(x\right)\right)\frac{\partial^{2}u\left(x,t\right)}{\partial x^{2}}\right)\\ & & \;+\left(\rho_{c}b_{c}h_{c}+\rho_{a}b_{a}h_{a}\left(x\right)\right)\;\frac{\partial^{2}u\left(x,t\right)}{\partial t^{2}}=\;0 \end{array}$$

where *E_c_* and *E_a_* are Young's modulus of cantilever and attached mass, respectively. The *I_c_* denotes the cantilever moment of inertia while *I_a_* is the position‐dependent added mass moment of inertia. The density of the cantilever and added mass are denoted by ρ_*c*_ and ρ_*a*_, respectively. To solve Equation (1), we assume the harmonic transverse displacement satisfying the boundary conditions that the deflection and slope at the clamped end are both zeros.^[^ ^32^ ^]^ So, the beam deflection can be expressed as:

(2) $$u\;\left(x,t\right)=\sum_{n=1}^{\infty }A\varphi_{n}\left(x\right)e^{j\left(\omega_{n}t\right)}\;$$

where φ_*n*_(*x*) is the nth eigenmode shape of cantilever and ω_*n*_ is the nth eigenfrequency of the cantilever, *A* is the arbitrary value of the amplitude.

In this method, the eigenmode shape of the cantilever to satisfy the boundary conditions is expressed as:^[^ ^40^ ^]^

(3) $$\begin{array}{ccc} \varphi_{n}\;\left(x\right) & = & \sin\left(\frac{\beta_{n}x}{L}\right)-\sinh\left(\frac{\beta_{n}x}{L}\right)+\frac{\sin\beta_{n}+\sinh\beta_{n}}{\cos\beta_{n}+\cosh\beta_{n}}\\ & & \left(\cosh\left(\frac{\beta_{n}x}{L}\right)-\cos\left(\frac{\beta_{n}x}{L}\right)\right) \end{array}$$

where β_*n*_ for the first fourth eigenmode are 1.8751, 4.6940, 7.8547, and 10.9955.

Using the Galerkin discretization^[^ ^41^ ^]^ and equalizing the mean values of the cantilever flexural work and kinetic energy per oscillation cycle, the resonance frequencies of the cantilever can be obtained as:

(4) $$\omega_{n}=\sqrt{\frac{\int_{0}^{L}E_{c}I_{c}{\ddot{\varphi }}_{n}^{2}dx+\int_{L_{1}}^{L_{2}}E_{a}I_{a}{\ddot{\varphi }}_{n}^{2}dx}{\int_{0}^{L}\rho_{c}b_{c}h_{c}\varphi_{n}^{2}dx+\int_{L_{1}}^{L_{2}}\rho_{a}b_{a}h_{a}\varphi_{n}^{2}dx}}\;$$

where *L*_1_ and *L*_2_ are represented in Figure 1.

## 3 Results and Discussion

### 3.1 Simulation Result

First, we use our presented model to analyze the effect of the added particle's position, mass and length on the frequency tuning of the microcantilever, which is defined as the change of the ratio of higher‐order eigenfrequencies to the fundamental one.

**Figure 2:**
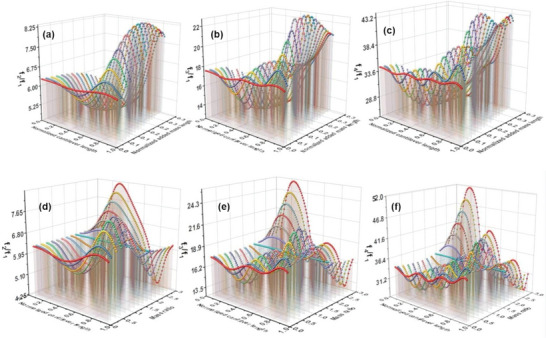
Simulation results for the higher order eigenfrequency over the 1st eigenmode. (a) ratio between 2nd and 1st (b) ratio between 3rd and 1st and (c) ratio between 4th and 1st eigenfrequency. In this simulation, the length of the deposited gold nanoparticles has been altered. (d) ratio between 2nd and 1st (e) ratio between 3rd and 1st and f) ratio between 4th and 1st eigenfrequency. In this simulation, the height of the deposited gold nanoparticles has been altered. The parameters of the cantilever for these simulations are adopted from the NuNano Scout 70 cantilever.

The simulation results to study the effect of the added mass length on the eigenfrequencies ratios are plotted in **Figure** **2**a–c. The ratio between the higher frequencies over the first natural frequency is plotted as a function of different cantilever positions. As the results show, the behaviors of the curves are different for each eigenmode and can be correlated with the mode shape curves.^[^ ^42^, ^44^ ^]^ From the analysis, we found that for certain cantilever positions and particular lengths of the added particle, simultaneous integer harmonics can be obtained (see Table S1, Supporting Information). Note that in our simulation we keep the height of the added particle fixed, so changing the length would lead to a change of the mass of the added particle as well.

These positions thus can be utilized experimentally to increase the signal‐to‐noise ratio of higher eigenmodes which can enhance the image resolution and accuracy of data extracted from multi‐eigenmodes of cantilever signals for quantification of materials properties. The effect of the mass by changing the height of the deposited nanoparticle on the eigenmodes is also studied and is plotted in Figure 2d–f where the ratio of different higher‐order eigenmodes and the first natural frequencies were plotted as a function of added nanoparticle mass. For this analysis, we keep the added particle length constant. Initially, at the low value of the mass loading on the cantilever, the frequency ratio behavior of the cantilever conforms to the previous situation where we varied the added particle length. Then, at a critical mass ratio (≈1.8 times the original cantilever mass), all the curve shape becomes flatter, and no wavy nature is observed. When the mass of the gold nanoparticles is further increased by increasing the particle height, the nature of the curves is reversed. This behavior can be correlated with the fact that when a foreign entity is attached to the cantilever, there would be an interplay between induced mass and stiffness changes. Depending on the shape, mass and position of the added particle as well as the cantilever mass itself, the resonance frequencies of the cantilever are varied. If the effect of mass changes is higher than changes in stiffness, then the natural frequency of the cantilever is reduced. On the other hand, when the added particle leads to a larger change in stiffness in comparison with the mass of the cantilever, it leads to the shift toward a higher natural frequency and subsequently the frequency ratio is varied.

To clarify the interplay between added mass and induced stiffness on the frequency response of the cantilever, we have performed a pertinent simulation. In this case, we utilized our simulation to study the changes in the first eigenfrequency of the modified cantilever by varying the mass of the added particles in two ways: 1) by altering the length and 2) by adjusting the height.

**Figure 3:**
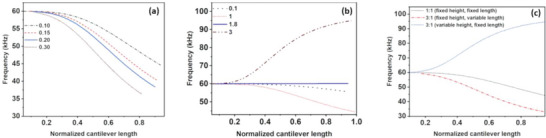
Variation of 1st eigenfrequency of the modified cantilever as a function of normalized cantilever length by changing the mass of the cantilever by (a) variation of added mass length and (b) variation of added mass height. (c) Comparison between 1st eigenfrequency for the simulative model with fixed height, variable length; fixed length, variable height and fixed length, fixed height of added mass.

For the variation of mass by changing the length of the added mass while keeping the height fixed, we plotted the first eigenfrequency shift. It was evident that as the length of the added mass increased, the frequency shift became more negative, indicating the dominance of the mass effect when length is varied (**Figure** **3**a). Then, we fixed the length of the added mass and varied the mass by altering the height of the added nanoparticles, plotting the results with respect to the cantilever position (Figure 3b). In this case, we observed that initially, with an increase in height, the eigenfrequency decreased, indicating a negative frequency shift. At a critical height, the frequency shift approached zero, and further increases in height resulted in a positive frequency shift. This positive frequency shift indicated the dominance of stiffness over mass.

Subsequently, we plotted another simulation graph, where we depicted the variation of frequency shift under three conditions: 1) when the length and height were kept constant, 2) when the mass was increased threefold by triplicating the length of the added particle while keeping the height constant, and 3) when the mass was increased threefold by triplicating the height of the added particle while keeping the length constant (Figure 3c). This scenario demonstrated that the effect of stiffness can become a dominating factor when the height is increased for the same mass of the attached particle. The simulation results of higher eigenfrequencies are shown in Figure S1 (Supporting Information). To verify our proposed theory, we have performed an experiment as well (Figure S3, Supporting Information) which is explained in detail in the experimental section.

Furthermore, the frequency ratio of the 2nd and 1st eigenmodes for these two cases (Figure 3c) are plotted and represented in Figure S2 (Supporting Information). To probe the relative change, the frequency ratio of 1st and 2nd eigenmode for the modified cantilever with the same Au: cantilever mass ratio and a fixed length (mimicking the experimental condition) is also plotted in the same graph and considered as a reference. Interestingly, the response of the frequency in both cases behaves differently. For the case where the higher mass loading is achieved by increasing the length, the frequency ratio plot follows a similar trend as that of the reference plot. However, the frequency response in the case of height‐dependent mass loading is quite different than that of the reference as well as the varied length situation. In the previous section, we have described that the eigenmodes of a modified cantilever depend upon the interplay between induced mass and stiffness of the attached particle. In the first simulation, the effect of stiffness on the eigenmodes of the cantilever is non‐existent and thus follows the reference plot. For the second simulation, at higher heights, the effect of stiffness is prominent and even higher than that of mass effect and thus a reversed nature of the graph is witnessed. This can be correlated with the fact that the increase in the frequency is correlated with the higher stiffness than the mass of the modified cantilever. The stiffness of a cantilever is proportional to the moment of inertia of the cantilever beam.

As the moment of inertia is proportional to the cube of the height of the cantilever, according to Equation (4), the increase in the nanostructured height would bring significant increase in the stiffness of the cantilever. So, as the stiffness effect is responsible for the positive frequency shift, the increase in height plays a significant role.

It means the changes in stiffness and subsequently the resonance frequencies of the modified cantilever are a function of the geometry of the added particle. This finding can explain why the resonance frequencies of the cantilever show both positive and negative shifts when particles with the same mass are added to it.^[^ ^31^, ^32^ ^]^

The simulation results are further compared with the finite element model (FEM) using three cantilever materials: (a) a silicon nitride‐based cantilever (E = 310 GPa), (b) a pure gold‐based cantilever (E = 79 GPa) and (c) a silicon cantilever (E = 179 GPa). The comparative plots for all the eigenfrequencies using these cantilevers are represented in Figures S4–S6 (Supporting Information). The close agreement between the trends observed in the analytical model and the numerical simulation across different materials strongly supports and validates the applicability of our simulation.

### 3.2 Experimental Results

To examine the effect of added particles on the dynamics and frequency response of the microcantilever as well as the spatial resolution of multifrequency AFM, we modified the cantilever by adding Au‐nanoparticles on the cantilever surface through a combination of micro drop assisted dip‐coating approach.

The SEM images of the as‐synthesized Au‐nanoparticle are represented in Figure S7 (Supporting Information). The particle size is analyzed, and the particle distribution is also represented in the inset. The average diameter of Au‐nanoparticles is calculated as ≈40 nm and the aqueous nanodispersion of the Au‐nanoparticles is utilized to modify the cantilever. More details about dip coating are given in the materials and methods section.

To demonstrate the generality of our method, we have used two different cantilevers in our experiments: NuNano Scout 70 and FMV‐A (Bruker) cantilevers. The experimental results and details of analysis for the FMV‐A cantilever are given in supporting information (Figures S8,S9 and Table S2, Supporting Information).

**Figure 4:**
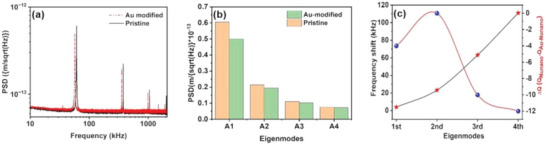
(a) The thermal noise spectra of pristine and Au‐modified cantilevers.(b) Variation of PSD with different eigenmodes for pristine and Au‐modified cantilevers, and (c) The variation of eigenfrequency shift and variation of the quality factor with eigenmodes.

The thermal spectra at room temperature in the air are collected for both pristine and Au‐modified NuNano Scout 70 cantilevers and are plotted in **Figure** **4**a. The natural resonance frequencies and the higher order flexural and torsional eigenmodes are recorded and the frequencies of 61, 378, 1054, and 2033 kHz are noted as the flexural frequencies for the 1st, 2nd, 3rd and 4th eigenmodes respectively and the peaks correspond to ≈1500 and ≈2000 kHz are found to be the torsional frequencies of the pristine cantilever. On the other hand, the flexural frequencies of the 1st, 2nd, 3rd and 4th eigenmodes of the modified cantilevers are found to be 56.75, 355, 991, and 1928 kHz, respectively. From the captured frequencies response, we can conclude that the addition of the Au‐nanoparticle to the cantilever leads to a decrease in natural resonance frequencies which is attributed to the dominancy of the mass change rather than the stiffness change of the modified cantilever.

In order to validate our theory regarding the directionality of frequency shift with changes in stiffness, we performed experiments by varying the mass of the cantilever. This was achieved by altering both the length and height of the added particle while measuring the corresponding shifts in the spring constant and frequency of the modified cantilevers. For a visual representation of these experimental results, please refer to Figure S3 (Supporting Information).

For the same mass, when we varied the length, we observed that the spring constant and the frequency shift were negative due to the added gold nanoparticles. Conversely, when the height was increased, the spring constant displayed a higher value compared to its pristine counterpart, and a positive frequency shift was observed, thus providing validation for our mathematical modelling and hypothesis.

By comparison between the results of simulations and experiments, we find the relation between the geometry of the added particle and the stiffness change of the cantilever. As in the simulations the mass of the added particle changes by length and height, for the case of the same added mass, we discover the sensitivity of stiffness to the height is higher than the length of added particle.

The spring constants of both the cantilevers at the flexural eigenmodes are measured and tabulated in **Table** **1**. The spring constants of the modified cantilevers at all the eigenmodes are lower compared to their pristine counterpart. It proves our hypothesis that in our modified cantilever, the effect of the mass changes is more dominant than the stiffness changes and explains why the eigenfrequencies are decreased.

**Table 1:** The spring constant of both the cantilevers at 1st, 2nd, 3rd and 4th eigenmodes.

1st eigenmode		2nd eigenmode		3rd eigenmode		4th eigenmode	
Pristine	Modified	Pristine	Modified	Pristine	Modified	Pristine	Modified
1.35 Nm^−1^	1.18 Nm^−1^	49.82 Nm^−1^	46.14 Nm^−1^	408.35 Nm^−1^	357.92 Nm^−1^	1044 Nm^−1^	947 Nm^−1^

The difference in the eigenfrequencies and the quality factor between the two cantilevers are represented in Figure 4b. A blue shift in frequency for all of the eigenmodes indicates that the Au‐nanoparticle deposition results in the reduction of the cantilever's natural eigenfrequencies. The shift is higher at a higher eigenmode indicating that the effect of added mass is more prominent at higher eigenfrequencies which agree with our analytical model and simulation. The energy dissipation in a cantilever can be monitored by examining the quality factor of the cantilever, and a higher quality factor corresponds to a lower energy dissipation thus a higher quality factor is beneficial for a better frequency resolution and imaging. From the graph in Figure 4b, it is observed that the gold‐modified cantilever has a higher quality factor as compared to its pristine counterpart. The thermal noise amplitudes of the eigenmodes (known as power spectral density or PSD) for both the cantilevers at all four flexural eigenfrequencies are plotted in Figure 4c. The amplitudes for all the eigenfrequencies of the pristine cantilevers are higher than that of the modified cantilever and the PSD value is maximum for the amplitude corresponding to the 1st eigenmode. Then gradually the PSD values decrease with higher eigenmodes. The same behavior can be observed for the Au‐deposited cantilever. However, the difference between the PSDs of pristine and modified cantilevers follows an opposite trend and a minimal difference in PSDs is observed for the 4th eigenmodes of the cantilevers. The fact that although the amplitude of the first eigenmode of the modified cantilever is much smaller than the pristine one and comparable amplitudes at higher eigenmodes indicates that the amplitudes at higher modes do not dissipate completely and the signal‐to‐noise ratio has been improved significantly with the Au‐deposition in the modified cantilever. The frequency ratio of higher modes concerning the first eigenmode is calculated for both cantilevers. An incremental frequency ratio for the modified cantilever can be observed and the ratio between the 4th and the 1st gives an integer value (34 times than the first frequency) which significantly improves the fourth mode performance in imaging and characterization.

To test the imaging performance of the modified cantilever, we have performed measurements using tapping mode AFM and bimodal AM AFM. Imaging of PS‐PMMA on a glass slide and HOPG samples are first carried out in air using the ac tapping mode. The reason why we chose PS‐PMMA and HOPG is to study the performance of the modified cantilever for different stiffness as well as both polymer and carbon‐based materials. For the measurements, the parameters are kept fixed for the imaging of PS‐PMMA using both the cantilever. The set point of 0.4 V, the scan rate of 3 Hz, and an integral gain of 50, with a scan size of 10 µm x 10 µm are set for the imaging. It is worth mentioning that we optimized the imaging parameters for the pure cantilever at the first eigenfrequency and then utilized these parameters for imaging with the other eigenmodes of both pristine and modified cantilevers.

**Figure 5:**
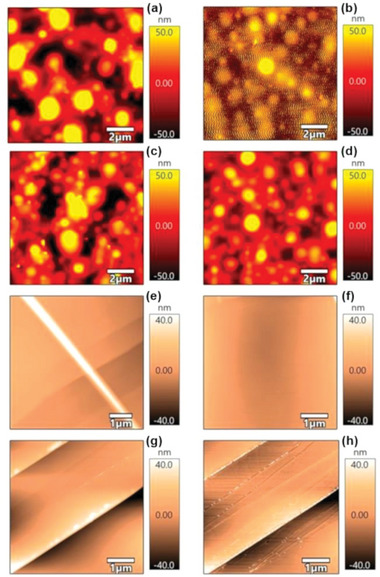
The AFM tapping mode images of PS‐PMMA and HOPG samples. Figure a–d represents the image of PS‐PMMA imaged with (a) pristine cantilever at the 1st eigenmode, (b) pristine cantilever at the 4th eigenmode (c) Au‐modified cantilever at the 1st eigenmode, and (d) Au‐modified cantilever at the 4th eigenmode. Figure e–h represents the imaging for HOPG where (e) pristine cantilever at the 1st eigenmode, (f) pristine cantilever at the 4th eigenmode (g) Au‐modified cantilever at the 1st eigenmode, and (h) Au‐modified cantilever at the 4th eigenmode used.

The tapping mode in the air for the 1st and the 4th eigenmode frequencies for the pristine and the modified cantilevers are obtained and represented in **Figure** **5**. It can be seen from the figure that the best resolution image is obtained for the pristine cantilever at the 1st eigenmode (natural frequency). This is expected as we set the imaging parameters for the first mode of the pristine cantilever as commonly the air tapping mode imaging is in general acquired at this condition.

The resolution is very poor when the imaging is carried out at the 4th eigenmode of the pristine cantilever and the features of the PS‐PMMA films are not prominent. Contrarily, the images acquired by the Au‐modified cantilever at the resonance frequency are not at par with the pristine cantilever. However, image quality and resolution have improved significantly for the image collected at the 4th eigenmode of the modified cantilever. This enhancement in the resolution can be correlated with the modified 4th eigenmode which is now converted into a harmonic frequency of the modified cantilever's natural frequency. To verify the improved imaging performance of the modified cantilever at harmonic frequency, imaging in the air using tapping mode on the HOPG sample is also carried out where the identical parameters of the set point of 0.4 V, the scan rate of 3 Hz, an integral gain of 50, with a scan size of 10 µm x 10 µm are set. The imaging of the HOPG has also followed the same trend where for the pristine cantilever, the higher resolution image collected at 1st eigenmode loses its resolution at 4th eigenmode and for the modified cantilevers, though the 1st eigenmode image has an acceptable resolution, the image quality and resolution improves drastically for the 4th eigenmode (as an integer harmonic of natural frequency). To quantify the comparative resolutions of tapping mode images obtained for PMMA‐PS and HOPG using the pristine and Au‐modified cantilevers, comparative image analysis algorithms using entropy and EAV methods are employed and the normalized values (normalized according to the first eigenmode of the pristine cantilever) are tabulated in **Table** **2**. The details of the image analysis‐based quantification method are given in the materials and methods.

**Table 2:** The normalized quantitative image analysis results calculated from the images represented in Figure 4.

Sample	Entropy	Sharpness
HOPG_pristine_1	1.00	1.00
HOPG_ pristine _4	0.96	0.97
HOPG_modified_1	1.00	1.01
HOPG_modified_4	1.01	1.47
PS‐PMMA_ pristine _1	1.00	1.00
PS‐PMMA _ pristine _4	N/A	N/A
PS‐PMMA _modified_1	N/A	N/A
PS‐PMMA _modified_4	0.96	0.97

For HOPG, the values of both the parameters are found to be below the unity for the 4th eigenmode imaging of the pristine cantilever, indicating a poorer image quality. For the 1st eigenmode of the modified cantilever, the values are almost unity, indicating the Au‐modified cantilever can obtain a similar resolution as the 1st mode of pristine one for HOPG. However, the values are greater than the unity for the Au‐coated cantilever at 4th eigenmodes indicating a much‐improved resolution as compared to even the natural frequency of the pristine cantilever. This finding is particularly important, and it can be noted that by exciting the cantilever with its harmonic frequency through tuning the higher eigenmodes, a higher‐resolution image can be obtained. A similar statistical analysis is carried out for the images obtained for the PS‐PMMA sample. However, all the images obtained for the Au‐modified cantilever exhibit values less than unity. Thus, the comparison between the Entropy and Sharpness values of the modified cantilever for PS‐PMMA and HOPG shows better performance of the 4th mode of the modified cantilever for HOPG. It can be explained by the higher stiffness of HOPG which enhances the harmonics and provides more sensitivity by using higher‐order eigenmodes of cantilevers.^[^ ^45^, ^46^ ^]^ As explained in the mentioned references, depending on the sample and cantilever stiffness, at the specific effective stiffness, the specific eigenmode can provide higher sensitivity in imaging. So, as HOPG is stiffer than PS‐PMMA, using a higher eigenmode can provide higher resolution as for 4th eigenmode as seen in our analysis. It is important to mention that PS‐PMMA images obtained for certain eigenmodes, such as for the 4th eigenmode of the pristine cantilever and 1st eigenmode of the Au‐modified cantilever lack of details and show very poor features that the image analysis algorithm is unable to be applied to these images. The reason for better contrast for HOPG rather than PS‐PMMA using 1st eigenmode of the modified cantilever in comparison with the 1st of the pristine one can be explained by the reduced frequency and spring constant of the modified cantilever.

**Figure 6:**
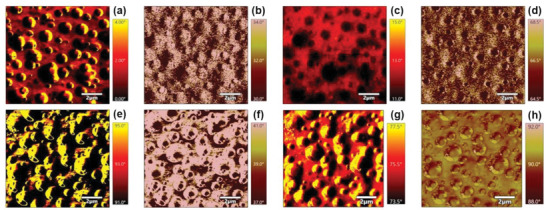
The bimodal Dual AC AFM 1st and 2nd phase mapping images of PS‐PMMA. Figure a–d represent the (a) first phase and (b) second phase image mapped with 1st‐2nd eigenmode of the pristine cantilever,(c) first phase and (d) second phase image mapped with 1st‐2nd eigenmode of Au‐modified cantilever. Then Fig e‐h demonstrates the (e) first phase and (f) second phase image mapped with 1st‐4th eigenmode of the pristine cantilever and (g) first phase and (h) second phase image mapped with 1st‐4th eigenmode of Au‐modified cantilever.

The bimodal amplitude modulation (AM‐AM) AFM technique is then introduced to explore the effect of higher‐order eigenmode tuning on the imaging quality of the polystyrene‐polymethylmethacrylate (PS‐PMMA) block co‐polymer assembly deposited on the glass slide. The imaging parameters are set at a setpoint of 0.55 V, a scan rate of 1 Hz, integral gain of 50, with a scan size of 10 µm x 10 µm. The AM‐AM images for the pristine and Au‐modified cantilevers for the 1st‐2nd and 1st −4th eigenfrequencies are collected. The first and the second phase mapping in the air for the pristine and Au‐modified cantilevers are represented in **Figure** **6**. For the pristine cantilever, the image quality is better for the 1st‐2nd eigenfrequency. The phase mapping collected for the 1st–4th eigenmodes lacks clarity and the resolution is inadequate for further analysis. In the case of the modified cantilevers, though the resolution for the phase mapping obtained at the 1st‐2nd eigenmode is relatively poorer, a much‐improved image quality, less distortion and detailing can be obtained by the 1st‐4th eigenmode imaging. The phase difference of the optimal phase image obtained from the literature^[^ ^47^ ^]^ is also matches with the images obtained from the 1st‐4th eigenmode of the modified cantilever. The detailed cross‐sectional analysis of second phase, imaged with the 1st‐ 2nd and 1st‐ 4th eigenfrequencies are represented in Figure S10 (Supporting Information). This is attributed to the fact that the 4th eigenmode matches the integer harmonics and a better coupling between the two eigenmodes is primarily responsible for the improved image quality.

As a summary, we have developed a theoretical and simulative model to analyze the effect of added mass on the frequency response of cantilevers and subsequently through the positioning of added practice, tune the ratio of higher eigenmode to the first resonance of cantilever to an integer value. In our study, we investigated how the cantilever frequency response is affected by the mass, length, and height of the attached particle, as well as its position on the cantilever. Our results indicate that increasing the height of the attached particle, rather than its length, leads to higher stiffness of the modified cantilever, even with the same attached mass.

To achieve specific changes in the cantilever's frequency response, we employed simulations based on our analytical model. By carefully selecting the appropriate mass and geometry for the attached particle, we were able to tune the cantilever's frequency response. This tuning allowed us to modify the eigenfrequencies to become integer multiples of the fundamental frequency, as detailed in Table S1 (Supporting Information). As a result of this coupling with the harmonics of the first mode, the specific eigenfrequencies exhibited an increase in the signal‐to‐noise ratio (SNR). Furthermore, using the dip coating approach, we added gold nanoparticles to the cantilever to improve its 3rd (FMV‐A cantilever) and 4th (NuNano Scout 70 cantilever) eigenmode response. Using measurements of the 3rd and 4th eigenmode response of both tapping mode and bimodal AFM, we have shown a significant improvement in image quality and resolution. Our results can open a path to not only improve the multifrequency AFM operation in either imaging or characterization but also can explain the attachment mechanism of nano/bioparticles on the dynamics response of cantilevers in sensing applications.

## 4 Experimental Section

### Chemicals and Materials

Gold (III) chloride trihydrate (HAuCl_4_,3H_2_O), citric acid, and sodium hydroxide (NaOH) of AR grade were purchased from Sigma–Aldrich (Merck, UK). All the aqueous solutions were prepared using ultrapure deionized water (Millipore Milli‐Q system) having a resistivity of 15 MΩ. HOPG was purchased from MikroMasch (NanoAndMore, USA).

### Au‐Nanoparticle Synthesis

The gold nanoparticle was synthesized from HAuCl_4_ using a citrate‐mediated thermal reduction method, already mentioned in the literature.^[^ ^48^ ^]^ In this method, 1 ml of 0.175 M L^−1^ aqueous citric acid solution was added into a 0.52 mM L^−1^ HAuCl_4_ solution and stirred constantly. To neutralize the solution pH, 1 ML^−1^ of an aqueous solution of NaOH was introduced after 12 s and was cooled. The final nano‐dispersed gold solution was collected and stored.

### Finite Element Analysis

The FEM simulation was performed using ANSYS software. In the numerical simulation, the geometry is generated and assembled in SOLIDWORKS and imported to ANSYS. STEP files prepared in ANSYS DesignModeler before application of boundary conditions. Fixed support is applied to one “end face” of the cantilever geometry.

For both analytical and numerical simulations the cantilever and added mass properties are:

Cantilever:

- Material = Silicon Nitride
- Density = 3170 kg/m3
- Young's Modulus = 310 GPa
- Poisson's Ratio = 0.27

Added mass:

- Material = Pure Gold
- Density = 193 200 kg/m3
- Young's Modulus = 79 GPa
- Poisson's ratio = 0.42

Furthermore, in our numerical simulations (Figures S5 and S6, Supporting Information), we changed Young's modulus of the cantilever to 179 and 79 GPa, respectively. Note that for the analytical simulation of Figures 2 and 3, we use the parameters of the NuNano Scout 70 cantilever.

### Preparation of PS‐PMMA Film

Polystyrene (PS, Mw = 290000 amu) and polymethyl methacrylate (PMMA, Mw = 350000 amu) (Sigma–Aldrich, Irvine, UK) were used to create the necessary 50%PS/50%PMMA weight‐to‐weight ratio (referred to herein as PS‐PMMA). To make a 3% casting solution of this demixed system 1.5 g of PS was mixed with 1.5 g of PMMA and 97 g (63.7 mL) of chloroform (119.38 g mol^−1^, Sigma–Aldrich, Irvine, UK) added. The solution was then placed on a vibrating plate in a stoppered flask for 24 h to ensure full PMMA dissolution and mixing.

Glass coverslips were placed in a beaker containing 99% isopropanol (IPA) (Sigma–Aldrich, Irvine, UK) and sonicated in an ultrasonic bath (Ultrawave Ltd., Cardiff, UK) for 15 min to remove any surface contamination before being rinsed with deionized water and placed into an oven operating at 70 °C to dry. Clean, dry substrates were placed in the vacuum chuck of an SCS G3P‐12 spin coating device (PiKem, Wilnecote, UK) and the PS/PMMA casting solution was applied dropwise onto the surface to create a fully coherent liquid layer. The spin program was then initiated which comprised ramping the chuck rotation up to 6000 rpm for three minutes. The film was then dried at room temperature before being stored in a desiccator.

Particle size distribution was determined by using scanning electron microscopy (SEM). SEM was carried out using a Hitachi SU5000 (Hitachi, Japan) microscope and images were acquired at 5 keV with a nominal spot size of 30 nm. Figure S11 (Supporting Information) shows a representative image captured at magnification x6000 which was utilized for particle size analysis. Image analysis was completed using the particle size analysis macro within ImageJ^[^ ^49^ ^]^ with an average particle size of 0.432 µm (minimum particle size = 0.152 µm; maximum particle size = 1.131 µm).

### AFM Methods

*AFM Probe*: The cantilever properties and the AFM imaging in the air were carried out using an Asylum research Oxford Jupiter AFM system. An uncoated Nu‐Nano Scout‐70 silicon cantilever (length 225 µm and width 30 µm, thickness 2.5 µm) and an FMV‐A silicon cantilever (length 230 µm and width 30 µm, thickness 2.5 µm) were used for the analysis and imaging.

### Modification of Cantilever using Au‐nano Dispersion

To deposit the Au‐nano dispersion at a specific location on the cantilever, we employed a combination of drop casting, dip coating, and transfer protocols. This methodology drew inspiration from the particle‐imprint method elucidated in the literature.^[^ ^50^, ^51^ ^]^ In our experiment, we first utilized the micro drop technique,^[^ ^52^ ^]^ applying a specific quantity of sub‐micron‐sized droplets with a diameter of roughly 40 nm and a known concentration in water onto a hydrophobic silicon surface. To regulate the added nanoparticle's mass, adjustments were made to the concentration and droplet volume. The mass of the desired Au‐nanoparticle was initially calculated from the simulation. In order to deposit this desired mass, initially a stock solution of higher concentration of Au‐nano dispersion was prepared and to obtain the specific concentration, the stock solution was serially diluted. For example, to deposit the Au nanoparticle on the NuNano cantilever, initially 20 ml of a 10 mM aqueous dispersion of Au nanoparticle was prepared. Then this stock solution was serially diluted to obtain an Au nano dispersion of 10 µM (diluted from 10 mM to 1 mM to 100 µM to 10 µM). Then using a precise micropipette 0.2 µl of this 10 µM dispersion was deposited on the hydrophobic silicon surface. Subsequently, the tiny droplet was transferred to the desired location on the cantilever by bringing the cantilever closer to the droplet and gently dipping the specific part of the cantilever. The use of a camera‐enabled programmable micro‐dip coater can make the deposition precise, controllable, and repeatable. Due to the hydrophobic nature of the cantilever surface, the gold nano‐dispersion assembled in a concentric area on top of the cantilever's body after a successful transfer. The cantilever was then dried at room temperature, and as the water molecules evaporated, the gold nanoparticles became attached to the cantilever.

The relative position of the mass deposition can be controlled by varying the angle of the cantilever concerning the droplet during the transfer process. Moreover, the length of the added mass can be altered by adjusting the volume of the nano‐dispersion, while the height can be changed by repeating the procedure multiple times.

To verify the accuracy of the deposition method in specific locations, it was performed ten repetitions of the deposition at targeted positions. The quantitative error analysis was conducted, and the average percentage error was calculated (the procedure is provided in the data analysis section of the manuscript). The obtained results revealed an average error of ≈1.3% of the total cantilever length. The error in the mass deposition is further quantified and the average percentage error is further calculated (the calculation was provided in the data analysis section of the manuscript and the calculation for the mass and error in the mass for the NuNano Scout 70 cantilever was represented in Table S3, Supporting Information). The as‐obtained results revealed an average error of ≈3%, indicating the reproducibility and reliability of the deposition protocol. The location and the corresponding average relative error (calculated from the average percentage error) of deposition were represented in Figure S12 (Supporting Information).

### AFM Measurements

Thermal noise vibrations of both the cantilevers were monitored to find the different eigenmodes of the cantilevers and the subsequent quality factor was calculated using the inbuilt‐provided software through the Lorentzian fitting method. The AFM images were acquired in tapping in air and bimodal dual AC in air mode. Quantitative image analysis and statistical methods were carried out to investigate the image quality.

### Statistical Quantification

In the entropy method,^[^ ^53^, ^54^ ^]^ the statistical feature of an image was analyzed to estimate the information regarding the richness of the image. The entropy (H) can be represented as:

(5) $$H\;=\;-\sum_{i\;=\;0}^{I}P\left(X_{i}\right)\log_{2}P\left(X_{i}\right)$$

where *P*(*X_i_*) is defined as the probability of a pixel having a grayscale value *X_i_* . I is the maximum grayscale value of the pixels.

(6) $$P\left(X_{i}\right)=\frac{\mathrm{histogram}\;\;c\mathrm{ounts}\;\left(X_{i}\right)}{\mathrm{sum}\;\left(\mathrm{histogram}\;\mathrm{counts}\right)}$$

The entropy of all the images was calculated using MATLAB and all the values are normalized and tabulated in Table 2. The entropy of the images also follows the same trends as that of the other statistical methods and the best image quality was obtained for the 1st eigenmode at 100% laser position.

The improved EAV algorithm^[^ ^55^ ^]^ was used to calculate the sharpness of all the images by evaluating the change of rate in a specific direction of the grayscale image. The sharpness for all the pixels was expressed as:

(7) $$EAV\;=\frac{\sum_{i=1}^{m\times n}\sum_{\alpha =1}^{8}\left|\frac{df}{dx}\right|}{m\times n}$$

where m and n are the image row and image columns respectively. α is the number of adjacent pixels, df is the change in grayscale and dx is the change in distance, and $\frac{df}{dx}$ is the gradient between adjacent pixels.

The algorithm was applied to all the images and the normalized values are tabulated in Table 2 and follow a similar pattern as the other image analysis tools.

### Data Analysis

All the data presented in this study were obtained through at least three measurements. To determine the error in the position of the added mass, the percentage error (δi) was first calculated as:

(8) $$\delta_{i}=\left|\frac{x_{m}-x_{c}}{x_{c}}\right|\;\times 100\%$$

where x_c_ is the center of the targeted deposition of the added particle and x_m_ is the measured center of the deposited nanoparticles.

To calculate the average percentage error ten depositions were conducted at the same locations. The average percentage error is then measured as:

(9) $$\delta_{\mathrm{avg}}=\frac{\sum_{i=1}^{10}\delta_{i}}{10}\;$$

The error in the mass deposition was calculated as the percentage error as Δ_*m*_:

(10) $$\Delta_{m}=\left|\frac{m_{\mathrm{dep}}-m_{d}}{m_{d}}\right|\;\times 100\%$$

where, *m_d_* (desired mass) is the desired deposited mass calculated from the volume and concentration and the deposited mass (*m_dep_*) is the mass of the added particle calculated from the effective mass (*m_e_*) of the cantilever.^[^ ^56^ ^]^

To calculate the actual deposited mass in our experiment we calculated the actual mass of the pristine and modified cantilever and then the actual deposited mass was calculated using the following relation:

(11) $$m_{\mathrm{dep}}=m_{\mathrm{am}}\;-m_{\mathrm{ap}}$$

where, *m_am_* and *m_ap_* are the actual mass of the modified and pristine cantilever respectively, calculated from the frequency and stiffness of the cantilevers.^[^ ^56^ ^]^

Five different added masses were analyzed and the average percentage error was further calculated as:

(12) $$\Delta_{\mathrm{avg}}=\frac{\sum_{i=1}^{5}\Delta_{i}}{5}\;$$

The AFM data was analyzed using the in‐built software provided by Asylum Research. MATLAB was utilized for simulations and quantitative image analysis. FEM analysis was performed using SOLIDWORKS and ANSYS software. The size of the nanoparticles, as well as the location and positions of the added mass, were measured with the ImageJ software. All graphs were plotted, and data analysis was carried out using the OriginPro graphing software.

## Conflict of Interest

The authors declare no conflict of interest.

## Supporting information

Supporting Information

## Data Availability

The data that support the findings of this study are available from the corresponding author upon reasonable request.
