# Supplementary material for: Tailored Microcantilever Optimization for Multifrequency Force Microscopy
Source: Adv Sci (Weinh). 2023 Oct 22;10(33):2303476. doi: 10.1002/advs.202303476 (PMC10667852; doi:10.1002/advs.202303476)
Supplement: Supplementary file 1 — Supporting Information [file ADVS-10-2303476-s001.pdf]

ADVANCED SCIENCE
Open Access

Supporting Information

for *Adv. Sci.*, DOI 10.1002/advs.202303476

Tailored Microcantilever Optimization for Multifrequency Force Microscopy

*Gourav Bhattacharya, Indrianita Lionadi, Andrew Stevenson, Joanna Ward and Amir Farokh Payam**

Supporting Information

Tailored Microcantilever Optimization for Multifrequency Force Microscopy

Gourav Bhattacharya, [1] *Indrianita Lionadi,* [1] *Andrew Stevenson,*[1] *Joanna Ward,* [1] *Amir Farokh Payam* [1]

[1] Nanotechnology and Integrated Bioengineering Centre (NIBEC), School of Engineering, Ulster University, Belfast BT15 1AP, United Kingdom

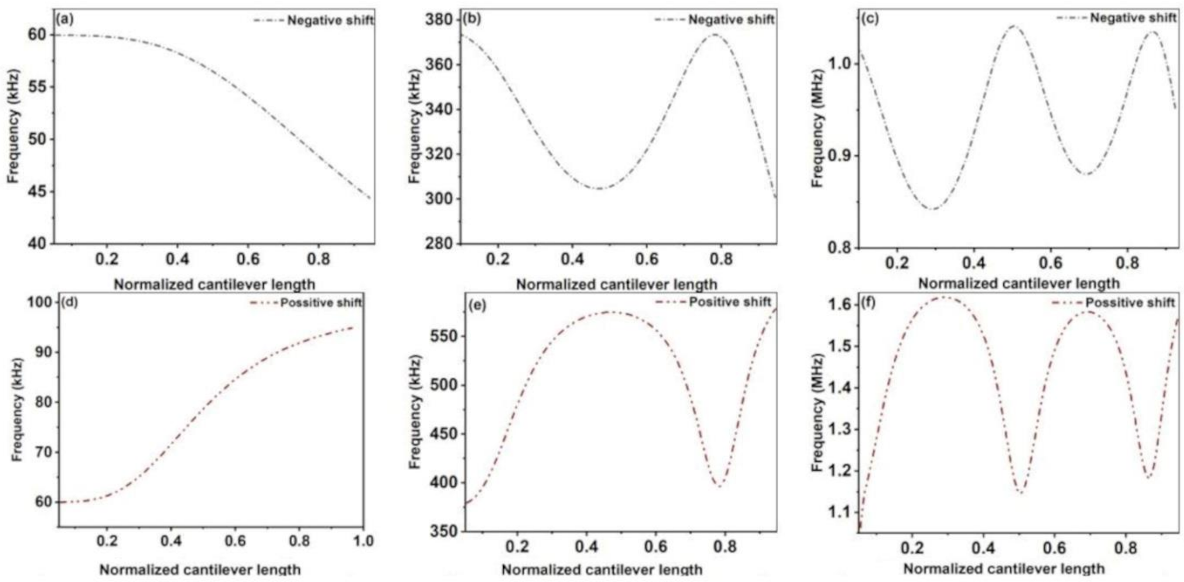

Figure S1 Comparison between 1st,2nd and 3rd eigenfrequency for the simulative model with positive and negative shift. Figure (a), (b), and (c) represents the negative frequency shifts for the 1st, 2nd, and 3rd eigenfrequency, respectively. Figure (d), (e), and (f) represents the positive frequency shifts for the1st, 2nd, and 3rd eigenfrequency, respectively. The eigenfrequencies of the 1st, 2nd, and 3rd eigenmodes of the pristine cantilever were 61 kHz, 378 kHz, and 1054 kHz, respectively.

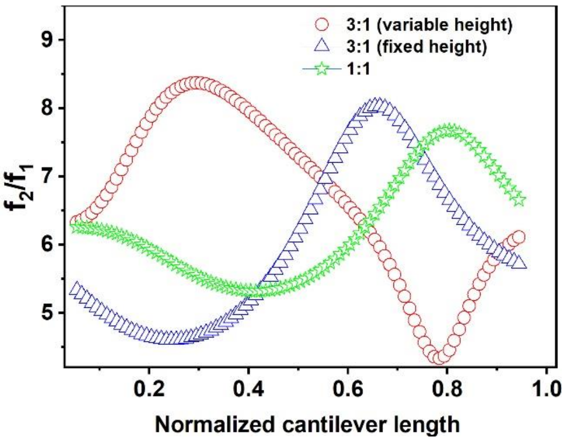

Figure S2 Comparison between frequency ratio between 2nd and 1st eigenfrequency for the simulative model with fixed height, variable length, fixed length, variable height and a fixed length and a fixed height of added mass.

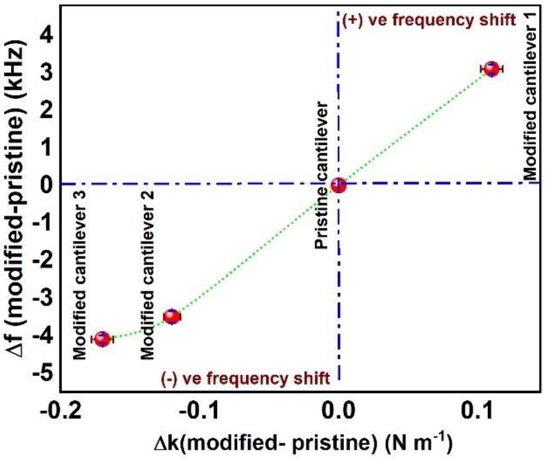

Figure S3 Variation of shift in spring constant and shift of first eigenfrequencies of different cantilevers with the same added mass for different particles with different length and height with respect to the pristine cantilever. The positive frequency shift is associated to the particle with less length and higher height.

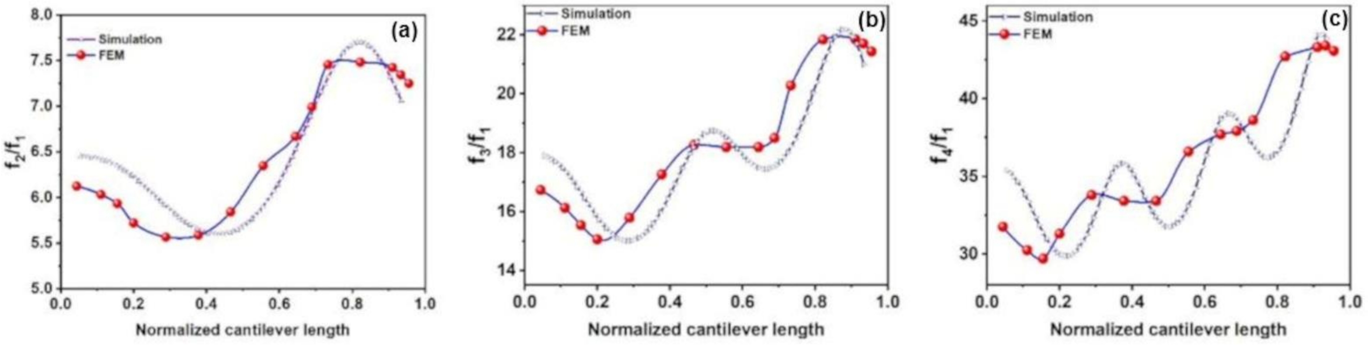

Figure S4 Comparison between Simulation and finite element model of a silicon nitride-based cantilever for the higher order eigenfrequency over the 1st eigenmode. (a) ratio between 2nd and 1st (b) ratio between 3rd and 1st and (c) ratio between 4th and 1st eigenfrequency.

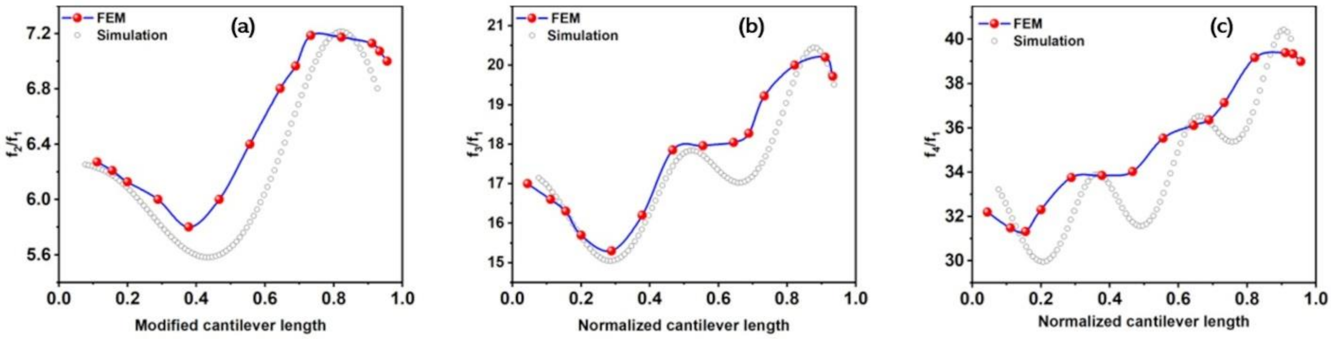

Figure S5 Comparison between Simulation and finite element model of a gold cantilever for the higher order eigenfrequency over the 1st eigenmode. (a) ratio between 2nd and 1st (b) ratio between 3rd and 1st and (c) ratio between 4th and 1st eigenfrequency.

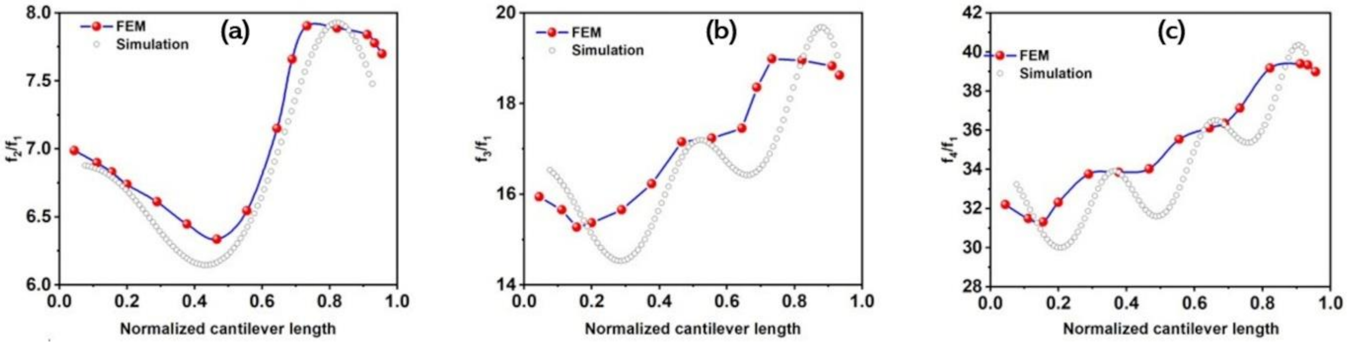

Figure S6 Comparison between Simulation and finite element model of a silicon cantilever for the higher order eigenfrequency over the 1st eigenmode. (a) ratio between 2nd and 1st (b) ratio between 3rd and 1st and (c) ratio between 4th and 1st eigenfrequency.

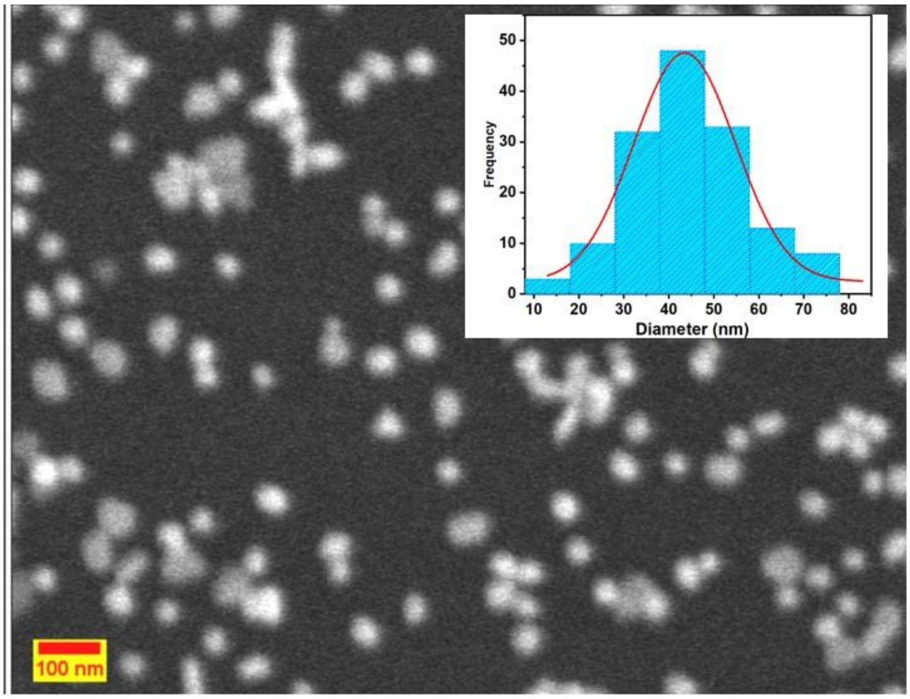

Figure S7 Scanning electron microscopy (SEM) image of the Au- nanoparticles. Inset exhibits the particle size distribution.

To show the generality of our proposed method, in our experiments, we have used an FMV-A cantilever as well.

The thermal spectra at room temperature in the air were collected for both the pristine and Au-modified FMV-A cantilevers, and they are represented in Fig. S8. The measurements encompassed the natural resonance frequencies and the higher-order flexural and torsional eigenmodes. For the pristine cantilever, the frequencies of 75.9 kHz, 477.3 kHz, 1344.9 kHz, and 2630.3 kHz were determined as the flexural frequencies for the 1st, 2nd, 3rd, and 4th eigenmodes, respectively.

Conversely, for the modified cantilevers, the flexural frequencies for the 1st, 2nd, 3rd, and 4th eigenmodes were found to be 67.8 kHz, 434.5 kHz, 1288.3 kHz, and 2420.1 kHz, respectively. From the frequencies captured in the response, we can infer that the addition of the Au-nanoparticles to the cantilever results in a decrease in natural resonance frequencies. This decrease is attributed to the dominant effect of the mass change rather than the stiffness change of the modified cantilever.

For the modified FMV-A cantilever, the ratio between the 3rd and the 1st eigenfrequency yields an integer value (19 times the first frequency), leading to a significant enhancement in the imaging performance in the 3rd eigenmode of the modified cantilever.

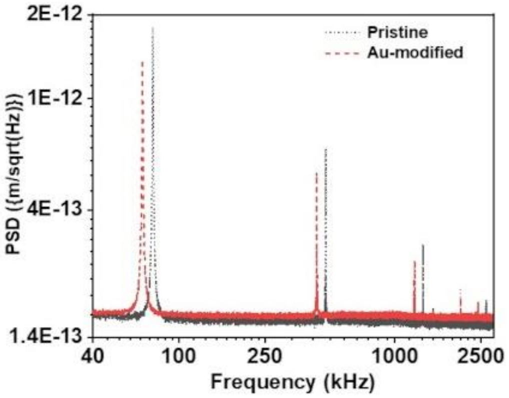

Figure S8 The thermal noise spectra of pristine and Au-modified FMV-A cantilevers.

To assess the imaging performance of the modified FMV-A cantilever, we conducted measurements using tapping mode AFM Imaging of PS-PMMA on a glass slide in air, employing the ac tapping mode. For these measurements, we maintained fixed parameters for imaging PS-PMMA using both cantilevers. Specifically, we set a set point of 0.6 V, a scan rate of 3 Hz, an integral gain of 50, and a scan size of 5 µm x 5 µm.

The tapping mode images for the 1st and 3rd eigenmode frequencies of both the pristine and modified cantilevers were obtained and presented in Supplementary Fig. S9.

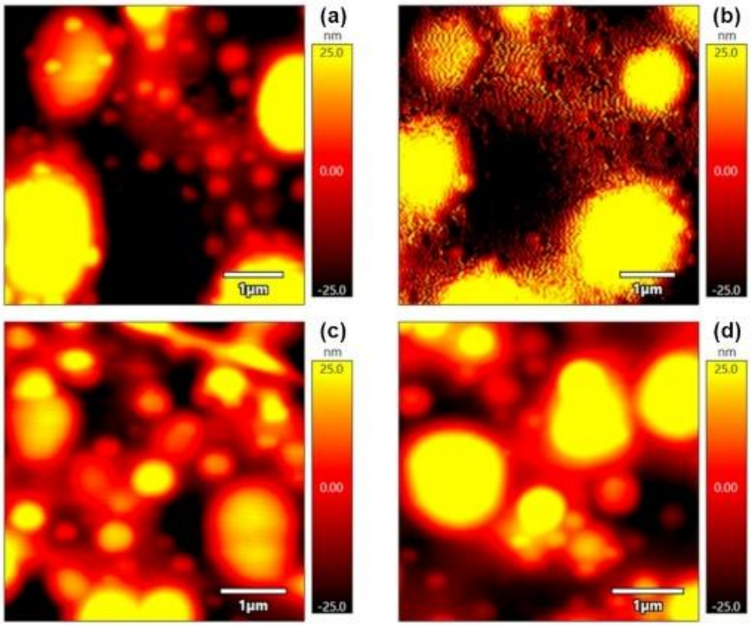

Figure S9 The AFM tapping mode images of PS-PMMA imaged with (a) pristine FMV-A cantilever at the 1st eigenmode, (b) pristine FMV-A cantilever at the 3rd eigenmode (c) Au-modified cantilever at the 1st eigenmode, and (d) Au-modified cantilever at the 3rd eigenmode.

The same was observed in the case of the NuNano Cantilever, the image obtained for the pristine FMV-A cantilever at the 1st eigenmode displayed good resolution, which is a typical outcome when imaging is performed in air tapping mode and we optimise the imaging parameters for this mode of pristine cantilever. However, the resolution significantly decreased when imaging was carried out at the 3rd eigenmode of the pristine cantilever, and the features of the PS-PMMA films were not well-defined.

On the other hand, the images acquired using the Au-modified cantilever at its resonance frequency were comparable to the 1st eigenmode image of the pristine cantilever. Notably, the image quality and resolution were markedly enhanced for the images collected at the 3rd eigenmode of the modified cantilever, surpassing those of the 3rd eigenmode of the pristine cantilever and approaching the level of the 1st eigenmode of the pristine cantilever.

This enhancement in resolution can be attributed to the modification of the 3rd eigenmode, which is now coupled with a harmonic of the modified cantilever's natural frequency. This harmonization facilitates improved imaging quality and resolution for the modified cantilever compared to its pristine counterpart. In this case as well, to quantitatively compare the resolutions of tapping mode images obtained for PS-PMMA using the pristine and Au-modified cantilevers, we utilized comparative image analysis algorithms based on entropy and EAV methods. The obtained values were normalized with respect to the first eigenmode of the pristine cantilever and are presented in supplementary Table S2.

Cross Sectional image analysis of bimodal dual ac scan

The study employed the NuNano Scout 70 cantilever, designed specifically for tapping mode operation in the air, for bimodal experiments. Resulted tapping mode images revealed the modified cantilever's superior image quality and resolution. The research aimed to compare image quality in bimodal dual AC mode using both unmodified and modified cantilevers on the PS-PMMA co-polymer blend. This blend consisted of PS cylinders with specific shapes within a PMMA matrix, necessitating consistent phase images to retain shape and properties. Uniform phases in PS and PMMA were vital, as phase variations could lead to measurement distortions and artefacts. Existing literature [1] established an optimal, and relatively small phase difference between PS and PMMA, cautioning against excessive phase differences that could lead to artefacts rather than enhanced resolution. Keeping all the points in mind, we have analyzed the second phase images and carried out the cross-sectional line profile of the phases and the results are provided in Figure S10. For the phase-2 of pristine cantilever imaged with

1st and 2nd eigenfrequency the phase image identifies unclear PS cylinder shapes, low signal-to-noise ratio, nearly 25° difference between PS and PMMA, and a substantial (~20°) phase variation in the PMMA region (Figure S10 a-d) which indicates a poor image quality. In the case of pristine cantilever imaged with 1st and 4th eigenfrequency the PS cylinder shapes are distorted, with an approximately 100° variation between PS and PMMA leading to changes in cantilever interaction from repulsive to attractive regime, and a significant (~15°) phase variation in the PMMA region signifying a non-reliable image quality. In case of the 2nd phase image for the 1st and 2nd eigenfrequency for the modified cantilever, though we observe a relatively low phase difference between PS and PMMA regions (~8°), and a relatively small phase variation specifically in the PMMA area (~5°), a low signal-to-noise ratio along with the distorted phase image of the PS regime makes the image quality poor. Finally for the second phase imaging for the modified cantilever imaged with 1st and 4th eigenfrequency the columnar PS phases are distinct, with a ~2.5° phase difference between PS-PMMA phases matching literature [1], and a minimal phase variation of under 0.5° in the PMMA region, indicating an accurate portrayal of the uniform PMMA phase in the sample and we can claim that as the phase structure is maintained, the phase difference is reasonable, a uniform phase of individual polymers is observed, and any noticeable distortions or noise is absent in the image, this particular image stands out as of the highest quality among those captured using the bimodal dual-ac scan.

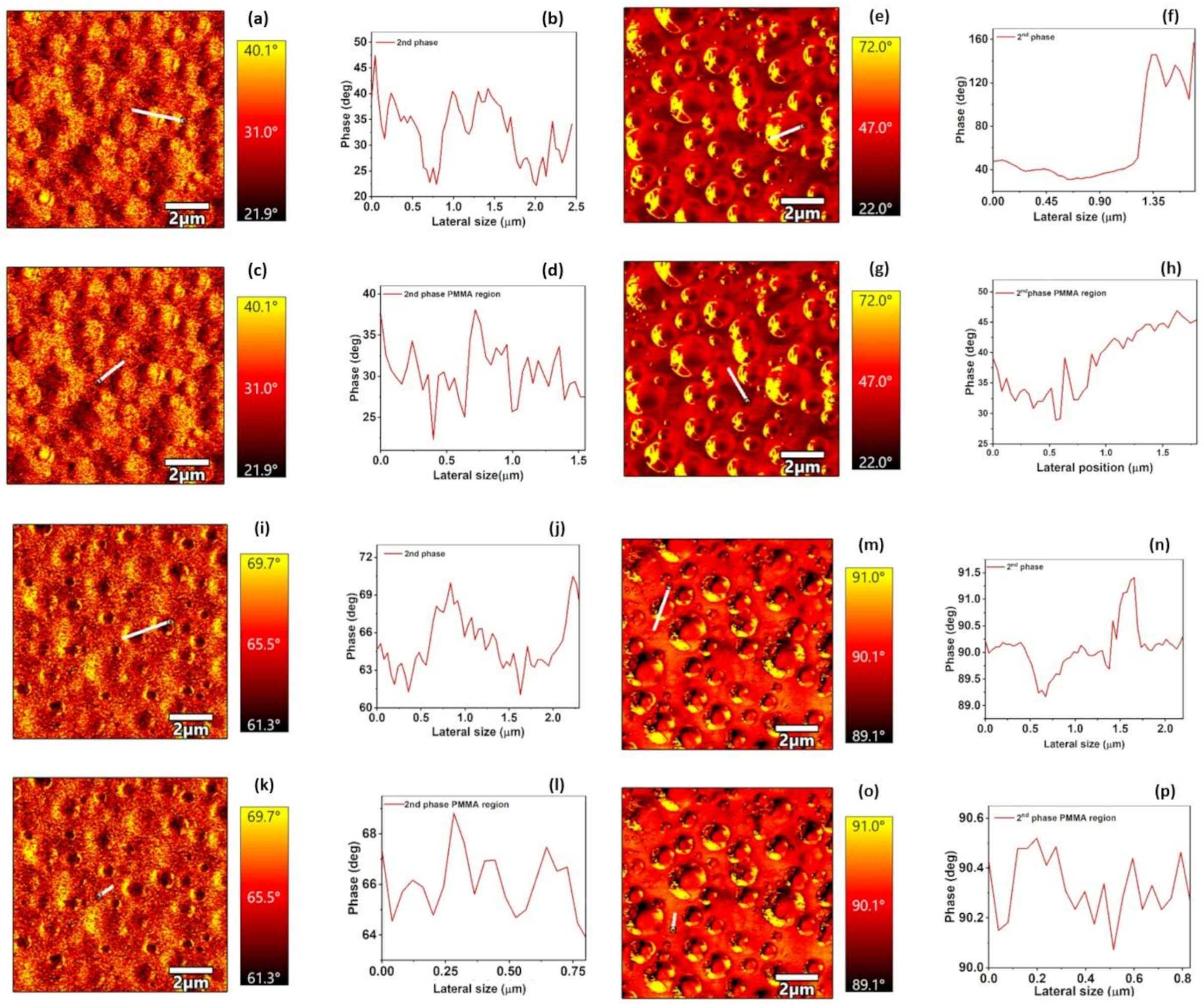

Figure S10 AFM (a) and (c) phase images of the PS-PMMA blend obtained by the pristine cantilever imaged with the simultaneous excitation of 1st and 2nd eigenmode of the pristine cantilever and (b), (d) are cross-section measured along the white line in panel a and panel c respectively, (e) and (g) phase images of the PS-PMMA blend obtained by the pristine cantilever imaged with the simultaneous excitation of 1st and 4th eigenmode of the pristine cantilever and (f), (h) are cross-section measured along the white line in panel e and panel g respectively, (i) and (k) phase images of the PS-PMMA blend obtained by the modified cantilever imaged with the simultaneous excitation of 1st and 2nd eigenmode of the Au-modified cantilever and (j), (l) are cross-section measured along the white line in panel i and panel k respectively and (m) and (o) phase images of the PS-PMMA blend obtained by the modified cantilever imaged with the simultaneous excitation of 1st and 4th eigenmode of the Au-modified cantilever and (n), (p) are cross-section measured along the white line in panel m and panel o respectively.

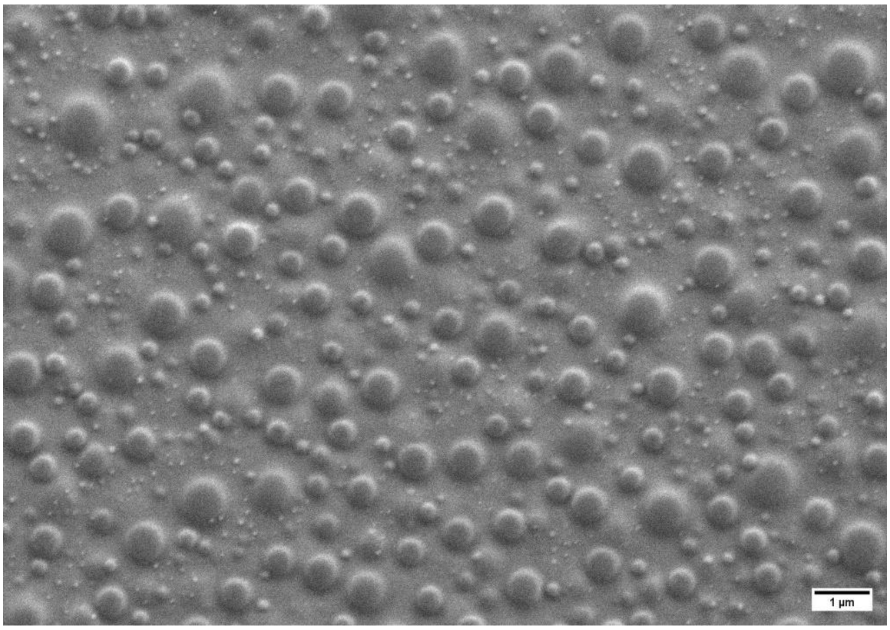

Figure S11 Scanning electron microscopy (SEM) image of the PS-PMMA film.

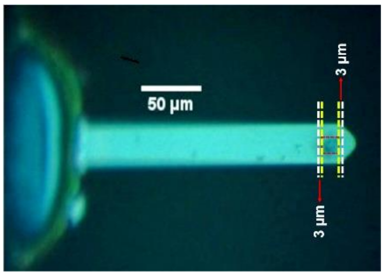

Figure S12 Optical image of the modified cantilever along with the corresponding error in the position of the deposited nanoparticles.

Table S1: Simultaneous tunning of eigenfrequency ratio to be an integer harmonic of the fundamental frequency.

Added mass length	Cantilever length	f_2/f_1	f_3/f_1	f_4/f_1
0.0222	0.4611	6	18	33
0.0444	0.8422	7	20	37
0.0889	0.1944	6	15	28
0.0889	0.4040	5.3	16	33
0.0889	0.9044	7.1	22	44
0.1111	0.6855	7	17	38
0.1333	0.1666	6	15	27
0.1333	0.8267	8	22	39
0.1556	0.3978	5	15	32
0.1778	0.2389	5.5	13	26
0.1778	0.1389	6	15	27
0.1778	0.3189	5	13	30
0.2000	0.1300	6	15	27
0.2667	0.8335	8	22	42
0.2667	0.6733	7	18	37
0.2889	0.5044	5	17	31
0.3333	0.4866	5	16	31
0.35556	0.4978	5	16	32

Table S2: The normalized quantitative image analysis result calculated from the images represented in Figure S9.

Sample	Entropy	Sharpness
PS-PMMA_ pristine FMVA _1	1.00	1.00
PS-PMMA_ pristine FMVA _3	N/A	N/A
PS-PMMA_ modified FMVA _1	0.98	0.99
PS-PMMA_ modified FMVA _3	1.00	1.01

Table S3: Calculation of added deposited mass and percentage error for the NuNano Scout 70 cantilever.

Sample	Stiffness (N/m)	Frequency (kHz)	Deposited mass from volume (ng)	Effective mass of cantilever (ng)	Real mass of cantilever after the particle is added (ng)	Added deposited mass (ng)	Percentage error (%)
NuNano Scout 70	1.35	61	0.394	9.19	38.98	0.386	2
Au-modified NuNano Scout 70	1.18	56.75		9.28	39.37		

[1] D. Mrđenović, D. Abbott, V. Mougel, W. Su, N. Kumar, and R. Zenobi, "Visualizing Surface Phase Separation in PS-PMMA Polymer Blends at the Nanoscale," *ACS Appl. Mater. Interfaces*, vol. 14, no. 21, pp. 24938–24945, 2022.
